# Targeting Serum Glucocorticoid-Regulated Kinase-1 in Squamous Cell Carcinoma of the Head and Neck: A Novel Modality of Local Control

**DOI:** 10.1371/journal.pone.0113795

**Published:** 2014-12-08

**Authors:** Henrik O. Berdel, Hongyu Yin, Jun Yao Liu, Karolina Grochowska, Christopher Middleton, Nathan Yanasak, Rafik Abdelsayed, Wolfgang E. Berdel, Mahmood Mozaffari, Jack C. Yu, Babak Baban

**Affiliations:** 1 Georgia Regents University, Augusta, GA, United States of America; 2 Palmetto Health/University of South Carolina School of Medicine, Columbia, SC, United States of America; 3 Plastic Surgery Hospital, Chinese Academy of Medical Science & Peking Union Medical College, Peking, China; 4 Department of Medicine A, Hematology and Oncology, University Hospital Muenster, Muenster, Germany; Ohio State University, United States of America

## Abstract

**Purpose:**

The inhibition of serum glucocorticoid-regulated kinase-1 (SGK-1) has been found to decrease growth of colon and prostate cancer cells. The purpose of this study is to evaluate the therapeutic effect of SGK-1 inhibition in head and neck squamous cell carcinoma (SCC).

**Experimental Design:**

Human head and neck tumors (HTB41/43) were established in athymic mice. Growth rates between mice treated with vehicle (PBS) injection (group 1, n = 5), SGK-1 Inhibitor GSK 650394 (group 2, n = 6), systemic cisplatin (group 3, n = 6), and a combination of SGK-1 Inhibitor and cisplatin (group 4, n = 6) were compared using repeated measures one-way ANOVA with Newman-Keuls Multiple Comparison Test. Tumor cells were subsequently submitted to further analyses.

**Results:**

At the end of the experiment mean tumor sizes were 122.33+/−105.86, 76.73+/−36.09, 94.52+/−75.92, and 25.76+/−14.89 mm^2^ (mean +/− SD) for groups 1 to 4. Groups 2 and 3 showed decreased tumor growth compared to controls (*p*<0.001). Group 4 displayed even greater growth suppression (*p*<0.0001). Importantly, group 4 fared better than group 3 (*p*<0.001). CD44 expression was reduced in group 2 (*p*<0.05), and to an even greater extent in groups 3 and 4 (*p*<0.0025). A trend towards reduction of HER 2 expression was noted in group 4.

**Conclusions:**

SGK-1 inhibition suppresses tumor growth, and in combination with systemic cisplatin exceeds the effect of cisplatin alone. Decreased expression of CD44 and HER 2 implies depletion of tumor stem cells, and less tumorigenicity. SGK-1 inhibition represents a potential modality of local control for palliation in advanced cases.

## Introduction

Most of the head and neck cancers (70–80%) are locally advanced upon diagnosis [1]. In the 1990s, the 5-year survival rate of these patients was reported as only 40% [2]. More than 50% of patients who succumb to the disease only have loco-regional failure, and close to 90% of patients with distant failure also have persistent loco-regional disease [3]–[5]. While the mainstay of treatment remains surgical excision, loco-regional control and survival has been improved with the addition of chemotherapy to postoperative radiation [6]–[8]. Cisplatin as the standard chemotherapeutic treatment agent is however associated with considerable side effects such as nephrotoxicity [9]. Recent evidence suggests multi-agent chemoradiotherapy to avoid the toxicity of single agents [10], [11]. Still, cancer treatment has been negatively impacted by the fact that classical chemotherapeutic drugs are characterized both by poor specificity and development of resistance [12]. The general damage created by these drugs triggers several mechanisms of apoptosis, some of which are rendered inoperative during oncogenic transformation and progression. This leaves a certain percentage of tumor cells resistant to chemotherapy-induced cell death [13].

With the progress in molecular targeted therapy of cancer, more specific targets have emerged that can be used to treat tumors by focusing on single or a set of molecules, or on molecular pathways crucial for tumor cell survival, but not as critical in normal cells. Over the past decade several kinases have been targeted. The serum glucocorticoid-regulated kinase-1 (SGK-1), a serine-threonine kinase well known for the regulation of renal sodium transport, has been described to support survival of tumor cells. Accordingly, tumor cell survival by an SGK-1 mediated mechanism has been implicated for IL-6-, IL-2, angiotensin II-, and androgen-receptor dependent cancer cells [14]–[19]. Furthermore, increased levels of SGK-1 expression have been found in many different tumors such as colon cancer, prostate cancer and non-small cell lung cancer [17], [20], [21]. Its precise function in these malignancies however remains unclear.

Our group has shown the anti-apoptotic potential of glycogen synthase kinase-3β (GSK3β) inhibition in ischemia-reperfusion experiments of the heart [22]. Inhibition of GSK-3β shows significant size reduction of infarction area. Furthermore, there is an exacerbating impact of pressure overload on the isolated ischemic reperfused heart due to GSK-3β-dependent increase in DNA injury and cell death via regulation of the mitochondrial permeability transition (MPT) pore [22]. As GSK-3β is inhibited by SGK-1 via phosphorylation [23], [24], we hypothesized that increased GSK-3β activity via local SGK-1 inhibition might cause increased regional cell death. In the literature, SGK-1 has been described as an anti-apoptotic kinase via several mechanisms. As such, it was shown to phosphorylate and thereby inactivate the proapoptotic forkhead transciption factor Foxo3a/FKRHL1 [25]. Furthermore, inhibition of GSK-3β by SGK-1 up-regulates oncogenic β-catenin [16], [26]. β-catenin represents the key intracellular component of the Wnt pathway, a powerful signaling cascade involved in development and cell-differentiation [27]. Importantly, β-catenin has been associated with the development of different cancers including tumors of the skin [28], [29]. GSK-3β phosphorylates the cytoplasmic pool of β-catenin at the N-terminus resulting in its ubiquitination and rapid degradation [30]. Therefore, SGK-1 deficiency decreases the cytoplasmic pool of active β-catenin through an abundance of active GSK-3β. Interestingly, SGK-1 has further been implicated in downregulating the pro-apoptotic transcription factor p53 by activating its ubiquitination and thereby degradation [14]. Finally, SGK-1 also inhibits apoptosis via the IκB/NFκB pathway by phosphorylating IKK, which in turn phosphorylates IκB leading to the loss of its inhibitory function on NFκB [17].

Cognizant of this background and our own results it was the goal of this study to test the growth suppressing and pro-apoptotic potential of SGK-1 inhibition in SCC of the head and neck. GSK 650394 is a specific, competitive inhibitor of SGK-1 that was manufactured for the treatment of prostate cancer, and found to decrease growth of androgen dependent prostate cancer cells [31].

As there is evidence that SGK-1 may confer resistance of breast cancer and colon carcinoma cells to chemotherapy [32], [33], and that SGK-1 inhibition may increase the toxic effect of chemotherapeutic drugs on tumor cells [17], we decided to test SGK-1 inhibitor GSK 650394 in comparison to cisplatin alone, and both drugs in combination. In addition to tumor growth, expression of the cancer stem cell (CSC) marker CD44, and the human epidermal growth factor receptor 2 (HER 2) was tested.

## Methods

### Squamous cell carcinoma cells

All animal procedures were performed in accordance with the approval of the Institutional Animal Care and Use Committee of Georgia Regents University. No human subject was involved in this study.

Head and neck SCC cell line samples (HTB41/HTB43) were purchased from ATCC (Manassas, VA, USA) and the cells were cultured according to the manufacturer’s instructions. Single cell suspension was achieved by harvesting the SCC cells from the culture followed by centrifugation (1500 rpm, 9 minutes). All sample incubations were in quadruplicate. Experiments were repeated twice for each primary culture tested.

### Subcutaneous tumor establishment in athymic mice

Athymic mice (Nude BalbC mice: n = 23) were purchased from Harlan Laboratories (Tampa, FL, USA), housed in barrier germ free area, and treated, retained and monitored according to the rules and regulation of the division of Laboratory Animal Services (LAS) at Georgia Regents University (GRU). The mice were injected subcutaneously with HTB41, and in a second experiment with HTB43 (2×10^6^ cells/animal). Subsequently, the size of the growing tumor was measured over time. Measurement entailed taking the longest diameter and a second diameter at a perpendicular angle to the first diameter. This was done to account for more accurate description of oval shaped growth in some tumors found in preliminary experiments, and performed equally for all 4 groups at every measurement taken. Subsequently, both diameters were multiplied to describe tumor size as a two-dimensional plane in the unit of square millimeters. Upon reaching the size of 4 mm in longest diameter, treatment with intratumoral PBS injection (group 1, controls), intratumoral SGK-1 Inhibitor injection (group 2), as well as systemic chemotherapy with cisplatin (group 3) and a combination of systemic cisplatin and intra-tumoral SGK-1 Inhibitor (group 4) was initiated. As the first tumor reached 15 mm in maximum diameter the mice were euthanized for further analysis.

### SGK-1 inhibition and systemic cisplatin chemotherapy

The SGK-1 inhibitor (GSK 650394) was purchased from Tocris (Bristol, UK). Groups 2 and 4 received SGK-1 Inhibitor by direct intra-tumoral injection twice weekly (20 microgram/mouse/injection) in a 150 microgram solution mixed with 1% phosphate buffered saline (PBS). The tumors of group 1 were injected with 150 microgram of 1% PBS. Groups 3 and 4 received systemic, intraperitoneal (IP) cisplatin therapy at 50% of the maximum tolerated dose (10 mg/mouse/week).

### Cytospin, Immunohistochemical staining, and quantification Protocols

Cytospin preparations from Head and Neck cancer cell line (HTB43) treated with and without SGK-1 inhibitor in vitro, were subjected to immunohistochemistry (IHC) staining for CD44 and Caspase 3 as described previously [21]. Further, formalin-fixed paraffin-embedded tumor tissue was cut in 4 µm sections and processed for immunohistochemical assessment of caspase 3 and CD44 according to previously described protocols [21]. All antibodies for immunohistochemistry analysis were purchased from BD BioSciences, USA. To quantify the expression of CD44 and Caspase 3 in tumor tissue, Metamorph computerized image analysis program (ImageJ software) was utilized. After obtaining the images, digital color thresholds distinguished the immunoreactive objects during initial automated steps of image analysis. Assignment of numerical values to stained areas ensued. This was followed by calculation of the ratio (in percent) of immunoreactive to nonreactive areas. For each tumor section, five zones (4 corners and one center) were analyzed and the average percent value recorded for each animal.

### Analytical Flow Cytometry

Tumor tissues from the experimental groups were sieved through a cell strainer (BD Biosciences, San Diego, CA), followed by centrifugation (1,500 rpm, 5 min) to prepare single-cell suspensions. Phenotypic analyses of cells were performed as described previously [34]–[36]. Briefly, samples were treated with reagents, and stained with fluorochrome-conjugated antibodies of anti human CD44 and HER-2 antibodies purchased from eBioScience (San Diego, CA). Next, cell samples were run through a four-color flow cytometer (FACS Calibur, BD Biosciences, San Diego, CA), and data were collected using CellQuest software. Samples were double-stained with control IgG, and cell markers were used to assess any spillover signal of fluorochromes; proper compensation was set to ensure the median fluorescence intensities of negative and positive cells were identical and were both gated populations. Gating was used to exclude dead cells and debris using forward and side scatterplots. As a gating strategy for each sample, isotype- matched controls were analyzed to set the appropriate gates. For each marker, samples were analyzed in duplicate measurements. To minimize false-positive events, the number of double positive events detected with the isotype controls was subtracted from the number of double-positive cells stained with corresponding antibodies (non-isotype control), respectively. Cells expressing a specific marker were reported as a percentage of total events.

### Statistical analysis

Data were expressed as means ± standard deviation (SD). The results were submitted to statistical analysis using Prism 5 software. An F-test was used to test for significantly effective pairing. Analysis of variance (repeated test one-way ANOVA) followed by post-hoc test (Newman-Keuls) was performed to test for statistical significance among growth rates, Caspase 3-, CD44-, and HER 2 expression between the different groups *in vivo*. For comparison of caspase 3, and CD 44 expression of HTB43 cell line incubated with vehicle versus SGK-1 Inhibitor *in vitro*, student’s t-test was utilized. *P*-values of less than 0.05 were considered as indicating significant differences.

## Results

### SGK-1 inhibition leads to caspase dependent cell death of HTB41 *in vitro*

**Figure 1:**
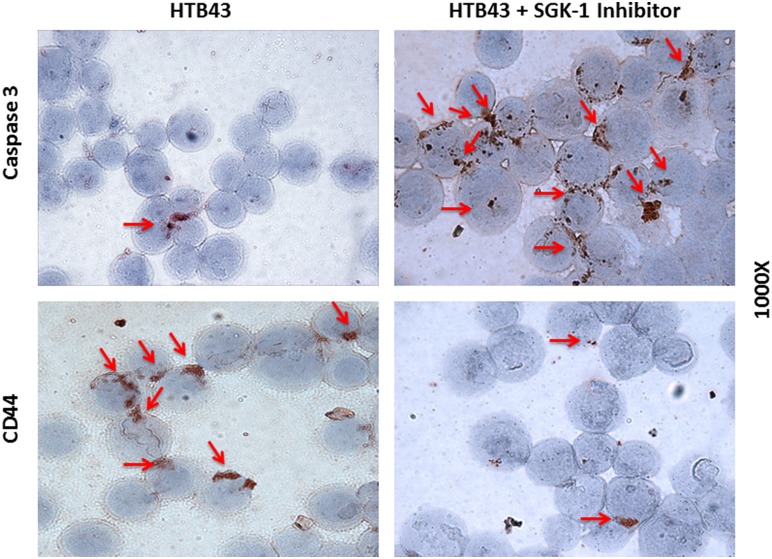
Cytospin assays of caspase 3 and CD44 expression of HTB cell lines *in vitro* showing markedly increased staining for caspase 3 after application of SGK-1 inhibitor, as well as decreased expression of CD44, however the latter did not reach statistical significance.

**Figure 2:**
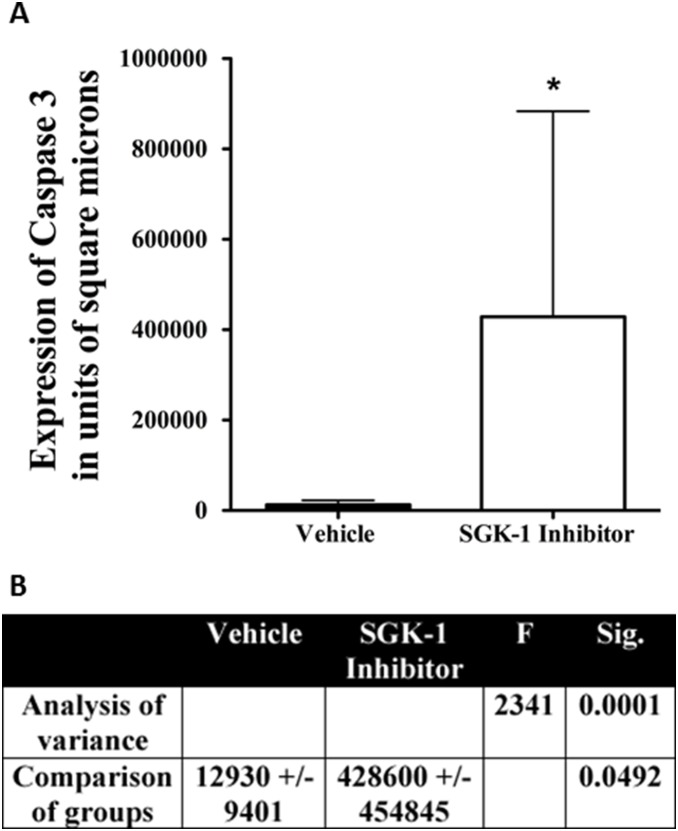
Bar graph depicting quantitative staining of caspase 3 expression after incubation with vehicle and SGK-1 inhibitor *in vitro* (A). Table illustrating results of analysis of variance, as well as student’s t test comparing vehicle and SGK-1 Inhibitor after incubation with HTB43 (**B**). Significant variance between the groups was given (*p* = 0.0001), and SGK-1 inhibition resulted in statistically significant higher caspase 3 expression than vehicle alone (*p* = 0.0492), indicating apoptosis as a possible mechanism of growth suppression in HTB41 treated with SGK-1 Inhibitor; Data in bar graph depicted as means +/− SD; Sig. = Significance.

In order to investigate whether SGK-1 inhibition attenuates the growth of head and neck squamous cell carcinoma cells, we initially treated incubated cell lines HTB41 and HTB43 for 72 hours with SGK-1 Inhibitor, and subsequently compared them to controls. An F-test indicated significantly different variances (*p* = 0.0001). SGK-1 inhibition resulted in statistically significant increased staining for caspase 3 on IHC implicating induction of an apoptotic mechanism (*p* = 0.0492, figs. 1, 2).

### SGK-1 inhibition exhibits significant growth suppression in SCC of the head and neck *in vivo*

**Figure 3:**
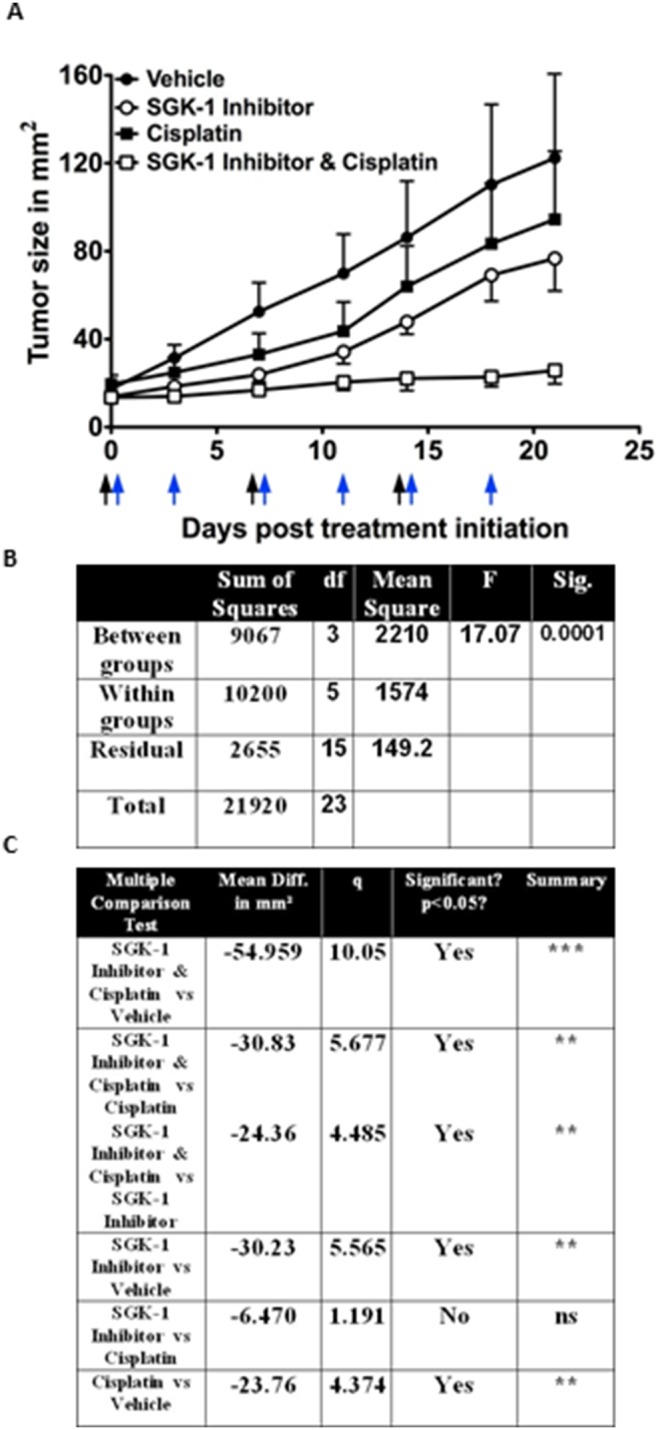
Growth Rates measured twice weekly after treatment initiation (A). Tumor sizes at the end of the experiment were 122.33+/−105.86 mm^2^ (mean +/− SD) in the control group, 76.73+/−36.09 mm^2^ in tumors treated with SGK-1 inhibitor, 94.52+/−75.92 mm^2^ in the Cisplatin group, and 25.76+/−14.89 mm^2^ with combination of local SGK-1 inhibition and systemic cisplatin. Black arrows indicate timing of systemic cisplatin-, and blue arrows timing of SGK-1 Inhibitor application. Repeated measures ANOVA for growth rates (**B**). Newman-Keuls Multiple Comparison Test for growth rates; df = degrees of freedom, Sig. = Significance; Diff. = Difference, q = studentized range value, ** = p<0.001; *** = p<0.0001 (**C**).

To further investigate the cell growth response we used a xenograft model, in which SCC cell lines were established subcutaneously. Animals were divided into four groups and treated with either intratumoral saline injection (group 1, controls), intratumoral SGK-1 Inhibitor injection (group 2), systemic intraperitoneal cisplatin (group 3), and a combination of systemic cisplatin and intratumoral SGK-1 Inhibitor injection (group 4). Tumor growth was measured twice weekly with an electronic measuring device, and growth rates compared at the end of the study (Fig. 3A). An F-test demonstrated significantly effective pairing between the groups (*p*<0.0001, Fig. 3B). Tumor sizes started differing just 3 days after initiation of treatment, and while the growth rate in the control group was fast, SGK-1 Inhibition showed a much slower acceleration than both controls and cisplatin, while interestingly the growth curve depicting tumor growth of combination of SGK-1 Inhibitor injection and cisplatin remained with the lowest increase in tumor sizes over the course of the experiment (Fig. 3A). At the end of the experiment tumor sizes were 122.33+/−105.86 mm^2^ (mean +/− SD) in group 1, 76.73+/−36.09 mm^2^ in group 2, 94.52+/−75.92 mm^2^ in group 3, and 25.76+/−14.89 mm^2^ in group 4. Comparison of the growth development via repeated test one-way ANOVA supported the clinical findings: Mice treated with SGK-1 Inhibitor faired significantly better than the controls (*p*<0.001, Fig. 2C). Although not statistically significant, SGK-1 inhibition showed greater growth suppression than cisplatin, which compared to controls achieved significant growth attenuation, as well (*p*<0.001, Fig. 3C). The combination of systemic cisplatin and SGK-1 Inhibitor injection was not only better than intratumoral vehicle injection (*p*<0.0001, Fig. 3C), but also showed a significant advantage over cisplatin therapy alone (*p*<0.001).

### Caspase dependent cell death is one possible mechanism involved in growth retardation of HNC by Cisplatin or Cisplatin & SKG-1 *in vivo*

**Figure 4:**
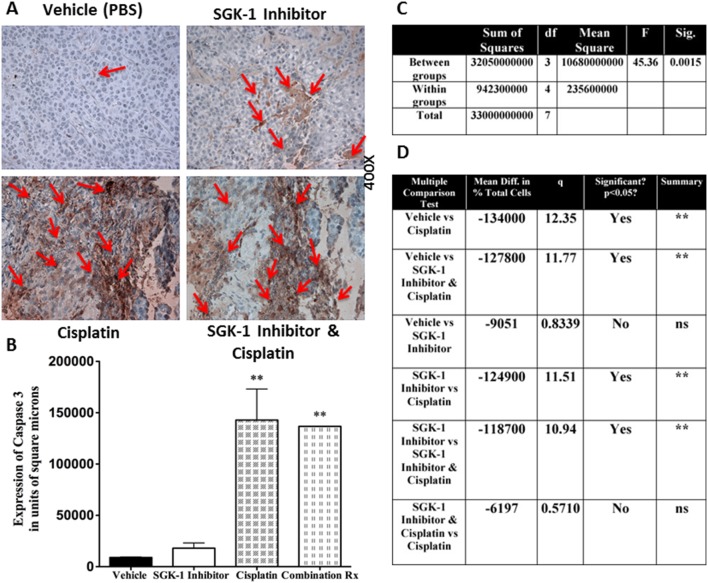
IHC stain for caspase 3 *ex vivo* (A). Bar graph showing results of quantitative staining for caspase 3 (**B**). One way ANOVA of caspase 3 expression (**C**). Results of Newman-Keuls Multiple Comparison Test for caspase 3 expression (**D**). Higher caspase 3 expression was found after local SGK-1 inhibition (17960.50+/−5183.80 square pixels) compared to controls (8910.00+/−568.51 square pixels). However, this result did not reach statistical significance, and power analysis revealed under-powering (79.10%). Larger sample sizes may help further clarify a possible difference between these groups. Combination of local SGK-1 inhibition and systemic cisplatin appeared to correlate with less caspase 3 expression (136697+/−209.30 square pixels) than cisplatin alone (142893.32+/−30249.32 square pixels). This result was not statistically significant (*p* = 0.82), and underpowered (8.80%). Less caspase 3 expression with combination of SGK-1 inhibition and cisplatin versus cisplatin alone may be due to completion of apoptotic mechanisms at the end of the experiment, or point towards an alternative synergistic mechanism of growth suppression with combination of SGK-1 Inhibitor and cisplatin other than caspase dependant apoptosis. Data reported as means +/− SD, df = degrees of freedom, Sig. = Significance; Diff. = Difference, q = studentized range value.

In order to confirm increased apoptosis as mechanism for depressed growth in tumors treated with SGK-1 inhibition, IHC with stains for the apoptotic marker caspase 3 were performed *in vitro* (figs. 1, 2), and *ex vivo* at the end of the experiment (Fig. 4). SGK-1 inhibition showed statistically significant increased staining for caspase 3 compared to controls *in vitro*, as seen in the cytospin slices shown in Fig. 1, and illustrated by bar graph and table in Fig. 2A and 2B, respectively. At the end of the experiment in vivo, tumor cells were submitted to IHC (Fig. 4), and the groups compared once again using ANOVA and Newman-Keuls multiple comparison test after quantification via ImageJ analysis. An F-test resulted in a statistically significant difference between groups (*p* = 0.0015, Fig. 4C). Caspase 3 expression was higher after local SGK-1 inhibition compared to controls (figs. 4A, B, and D). However, this result did not reach statistical significance. Power analysis between these groups revealed under-powering (79.10%), which may be one possible reason explaining this result. Both cisplatin and SGK-1 Inhibitor combined with cisplatin showed a significant increase in caspase 3 expression indicating the involvement of caspase dependant cell death in the antitumoral activity of cisplatin. Combination of SGK-1 inhibition and cisplatin therapy appeared to correlate with less caspase 3 expression than cisplatin alone (figs. 4B and 4D). Nonetheless, this result was not statistically significant (*p* = 0.82), and also underpowered (8.80%) in this study.

### SGK-1 inhibition depletes cancer-initiating cells

**Figure 5:**
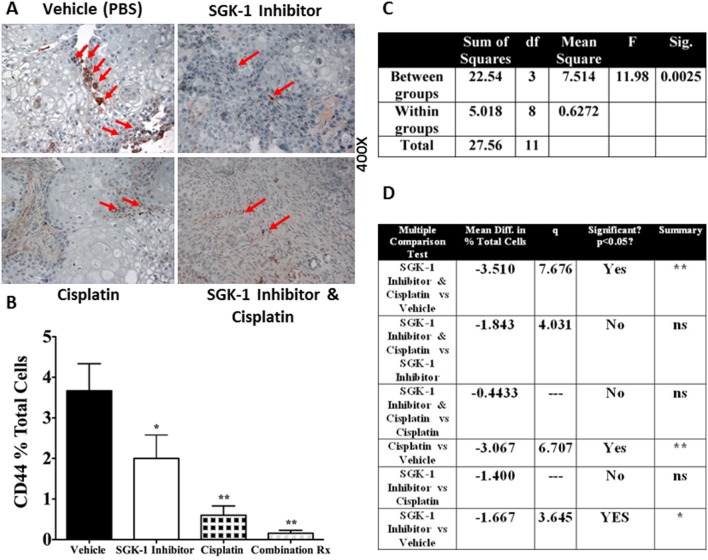
*Ex vivo* IHC staining for Caspase 3 (A). Bar graph depicting the results of ANOVA and post hoc test of the FACS analysis for CD44 expression (3 mice per group) in percent positive cells per 100 cells analyzed (**B**). One-way ANOVA for CD44 expression (**C**). Newman-Keuls Multiple Comparison Test for CD44 expression (**D**). While there was no statistically significant difference in CD44 expression between tumors of mice treated with Cisplatin vs Cisplatin and SGK-1 Inhibitor, power analysis revealed under-powering (44.10%). Higher sample sizes may be able to further clarify a possible statistically significant difference in CD44 expression between these groups; Rx = therapy, *p<0.025, **p<0.0025; Diff. = Difference, q = studentized range value.

**Figure 6:**
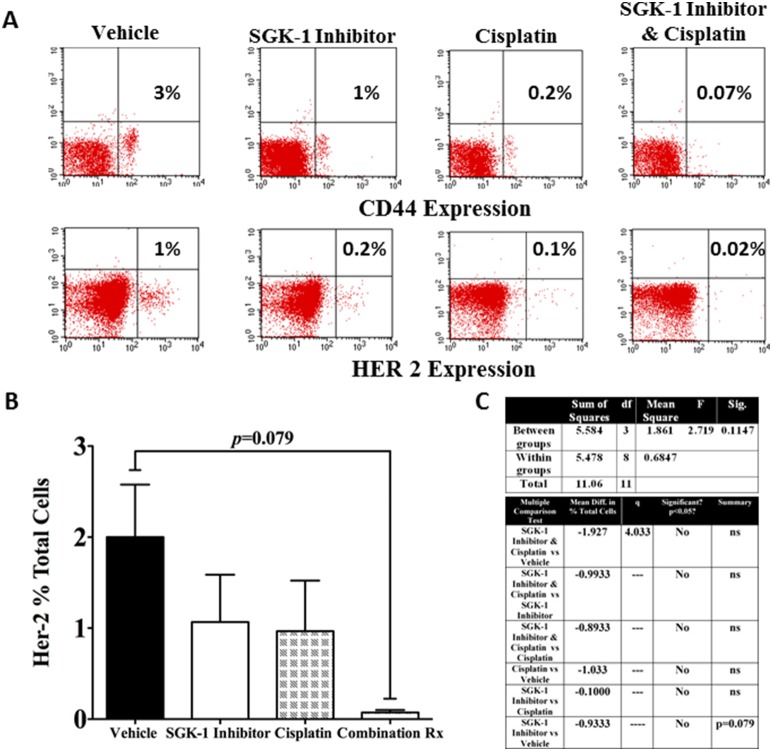
Example of FACS analysis showing CD44 and HER-2 expression (A). Bar graph illustrating the results of FACS analysis of HER-2 expression (3 mice per group) in percent cells per 100 cells analyzed; Rx = therapy (**B**). Results of one-way ANOVA of HER-2 expression including Newman-Keuls Multiple Comparison Test; Diff. = Difference, q = studentized range value (**C**, **D**). No statistically significant differences between the groups were found, however a tendency towards statistically significant reduction of HER-2 expression with combination of SGK-1 inhibition and cisplatin compared to controls was noted (power = 91.60%); df = degrees of freedom, Sig. = Significance; Diff. = Difference, q = studentized range value.

In order to investigate the effects of the different treatment modalities on malignant potential and propensity towards worse outcomes, we subjected tumor cells to FACS analysis of CD44, a marker for cancer-initiating cells [37]–[39]. *In vitro*, CD44 expression was reduced compared to controls (Fig. 1), but did not reach statistical significance. Combination of local SGK-1 injection and systemic cisplatin showed less expression of CD44 compared to cisplatin alone, however this result did not reach statistical significance, and was underpowered (44.10%). IHC staining for CD44 further substantiated the findings of FACS (Fig. 5A). An F-test revealed statistically significant differences between the groups (*p*<0.0025, Fig. 5C). CD44 was found to be significantly decreased by SGK-1 inhibition over controls (*p*<0.025), however to an even greater extent by systemic cisplatin (*p*<0.0025, figs. 5B and 5D). *In* *vivo* however, FACS of tumor cells of 3 mice after the end of the experiment showed a marked decrease in CD44 expression with SGK-1 inhibition (Fig. 6A).

### SGK-1 inhibition in combination with systemic cisplatin shows a tendency towards HER 2 reduction

As marker for migration and invasion [40] we submitted tumor cells to FACS analysis of HER 2 expression. An example of dot plots depicting HER 2 expression at the end of the experiment is shown in Fig. 6A. An F-test resulted in no statistically significant differences between the groups (*p* = 0.1147, Fig. 6C). HER 2 was reduced by SGK-1 inhibition, and even more so by cisplatin, however the most by combination of SGK-1 inhibition and cisplatin (figs. 6B and 6C). None of these results were statistically significant. Nevertheless, SGK-1 inhibition in addition to cisplatin revealed a tendency towards statistically significant reduction in HER 2 expression (*p* = 0.079, figs. 6B and 6C).

## Discussion

The limited success, high morbidity, and stress associated with repeated excision of large SCC of the head and neck characterize a high medical need of additional and alternative therapy options [6]–[8]. As systemic chemotherapy with cisplatin and/or 5-FU have limitations based on their toxicities, as well as limited response rates [9], [41], molecular targeted approaches have entered the fore. A primary example in SCC of the head and neck is cetuximab, an epithelial growth factor receptor (EGFR) inhibitor, which has been shown to improve outcomes of recurrent SCC of the head and neck after platinum based chemotherapy, as well as radiation [42]–[44]. Herein, we show for the first time that competitive inhibition of SGK-1 has potent antitumor properties in SCC of the head and neck, in particular when combined with cisplatin. Abundant literature has proven the apoptotic potential of SGK-1 inhibition by several different mechanisms [14], [16], [17], [25], [26]. Our group has just recently shown that SGK-1 inhibition is associated with disruption of the mitochondric membrane potential, and leads to apoptotic and necrotic cell death [45]. The *in vitro* findings of this study corroborate the apoptotic potential of SGK-1 inhibition, and for the first time show its clinical effect in SCC of the head and neck. Our analysis of caspase expression *in vivo* did not reach statistical significance for SKG-1 inhibition over controls, however this may be due to under-powering, or the fact that *in vivo* analysis for caspase 3 expression was performed at the end of the experiment, a point in time at which most apoptotic mechanisms may have already been completed. Importantly, in this study the combination of local SGK-1 inhibition and systemic cisplatin surpassed the growth suppressing effect of cisplatin alone, suggesting a mechanistic link that should be further investigated. Resistance to systemic chemotherapy mediated by SGK-1 has been published previously [16]. Moreover, Lang et al. have shown up-regulation of SGK-1 during ischemia, and stressed the importance of SGK-1 in ischemic tumor cells [15], [25]. Taking into account the previously published dependence of cisplatin treated squamous cell cancer on autophagy [46], it is tempting to speculate SGK-1 inhibition may play a role in this process, and increased cisplatin toxicity may result from a SGK-1 regulated attenuation of autophagic pathways. To evaluate for aggressive behavior and invasiveness, the expression of CD44 was analyzed. CD44 represents a marker for cancer initiating cells in HNC, and is associated with high tumorigenicity [37]–[39]. We were able to show that inhibition of SGK-1 significantly reduces CD44 expression. Combination of local SGK-1 Inhibitor injection and systemic cisplatin suppressed CD44 expression to an even greater extent. There was no statistically significant difference between SGK-1 inhibition in addition to systemic cisplatin and systemic cisplatin alone, however power analysis revealed under-powering. Although it is difficult to make this assumption, higher sample sizes may very well show a statistically significant result looking at these two groups individually. SGK-1 has further been described to enhance migration via actin cytoskeleton redistribution through down-regulation of vinculin phosphorylation [47]. Thus, we hypothesized SGK-1 inhibition may also affect migration and invasion of cancer cells, thereby potentially improving the outcome. In order to evaluate this additional and important mechanism, we tested the tumors for HER 2 expression. HER 2 is a cell surface protein frequently amplified in aggressive malignancies, and associated with migration and invasion of human head and neck cancer [40]. Interestingly, our results show a tendency of combination of local SGK-1 inhibition and systemic cisplatin to reduce HER 2 expression, although this result did not reach statistical significance. Further investigation may be necessary to further elucidate the relationship of SGK-1 and HER 2, and delineate a potential mechanism by which SGK-1 inhibition could reduce HER 2 expression in human head and neck cancer. Taken together, the results of growth attenuation, reduction in CD44 expression, and tendency towards reduced HER-2 expression propose that local SGK-1 inhibition in combination with systemic cisplatin may lead to growth attenuation, less migration, invasion, and malignant potential, and may allow for reduced cisplatin dosages in patients with increased cisplatin toxicity and better local disease control. There was no obvious increase in toxicity between the group treated with SGK-1 inhibition and cisplatin versus the group treated with cisplatin alone, as perceived by death or general subjective evidence of malaise in these animals. However, there were no objective measurements of toxicity (i.e. renal function) included in this study, and there is a chance that toxicity may be greater with combination of both drugs, even if SGK-1 is only used locally. Toxicity of local SGK-1 inhibition and in combination with cisplatin will need to be elucidated in additional studies. Overall the novel approach of SGK-1 inhibition in HNC should be evaluated further, as results of this murine model show great promise. As other authors have speculated, pathways such as ACT/PI3K could be up-regulated when SGK-1 is suppressed [48], leading to evasion of apoptosis. Ultimately, a better understanding of this mechanism and other pathways involved may lead to a potent anticancer treatment protocol that, while locally applied, and in combination with lower dosed systemic chemotherapy, will provide an excellent strategy of local control as alternative to increasingly morbid repeated resections and toxic high dose chemotherapy in palliative and/or neoadjuvant scenarios.
